# Fiber Clustering Acceleration With a Modified Kmeans++ Algorithm Using Data Parallelism

**DOI:** 10.3389/fninf.2021.727859

**Published:** 2021-09-01

**Authors:** Isaac Goicovich, Paulo Olivares, Claudio Román, Andrea Vázquez, Cyril Poupon, Jean-François Mangin, Pamela Guevara, Cecilia Hernández

**Affiliations:** ^1^Department of Electrical Engineering, Universidad de Concepción, Concepción, Chile; ^2^Department of Computer Science, Universidad de Concepción, Concepción, Chile; ^3^Université Paris-Saclay, CEA, CNRS, Neurospin, Baobab, Gif-sur-Yvette, France; ^4^Center for Biotechnology and Bioengineering, Santiago, Chile

**Keywords:** fiber clustering, white matter bundle, parallel computing, data parallelism, GPGPU—CUDA

## Abstract

Fiber clustering methods are typically used in brain research to study the organization of white matter bundles from large diffusion MRI tractography datasets. These methods enable exploratory bundle inspection using visualization and other methods that require identifying brain white matter structures in individuals or a population. Some applications, such as real-time visualization and inter-subject clustering, need fast and high-quality intra-subject clustering algorithms. This work proposes a parallel algorithm using a General Purpose Graphics Processing Unit (GPGPU) for fiber clustering based on the FFClust algorithm. The proposed GPGPU implementation exploits data parallelism using both multicore and GPU fine-grained parallelism present in commodity architectures, including current laptops and desktop computers. Our approach implements all FFClust steps in parallel, improving execution times in all of them. In addition, our parallel approach includes a parallel Kmeans++ algorithm implementation and defines a new variant of Kmeans++ to reduce the impact of choosing outliers as initial centroids. The results show that our approach provides clustering quality results very similar to FFClust, and it requires an execution time of 3.5 s for processing about a million fibers, achieving a speedup of 11.5 times compared to FFClust.

## 1. Introduction

In order to discover relevant information in tractography datasets, the research community has proposed several unsupervised approaches based on clustering algorithms to identify the white matter (WM) main structures based on shapes and streamline point positions. Some schemes used well known clustering algorithms such as spectral clustering (O'Donnell and Westin, 2007; O'Donnell et al., 2017), hierarchical clustering (Guevara et al., 2011b, 2012; Siless et al., 2018), label fusion clustering (Jin et al., 2014), and fuzzy c-means (Li et al., 2010). Recent methods use sampling, variation of distance metrics, multi-core parallelism, and different algorithm approaches and representations (Garyfallidis et al., 2012, 2014, 2016; Vázquez et al., 2020).

Several applications that use fiber clustering algorithms include multi or inter-subject clustering (Dodero et al., 2015; Huerta et al., 2020), WM atlas construction (Guevara et al., 2017; Román et al., 2017; Zhang et al., 2018), bundle segmentation based on atlases (Guevara et al., 2012; Jin et al., 2014; Labra et al., 2017), and connectivity-based cortical parcellations (Moreno-Dominguez et al., 2014). Some approaches combine clustering methods with anatomical information to identify known bundles (Li et al., 2010; Yoo et al., 2015; Garyfallidis et al., 2018). In addition, researchers use visualization tools for improving the understanding and interpretation of tractographies and segmented fiber bundles (Garyfallidis et al., 2012; Guevara et al., 2015; Combrisson et al., 2019). However, most of the clustering algorithms based on traditional clustering methods applied on large datasets are usually computationally expensive. Hence, recent works have proposed clustering schemes to improve computational time while maintaining high-quality results (Garyfallidis et al., 2012, 2014, 2016; Vázquez et al., 2020).

With the increase in data volumes and parallel architectures, parallel computing has become a powerful paradigm to improve performance in various applications. Building parallel clustering algorithms include different platforms and strategies. Some strategies use sampling (Guha et al., 2001), data partitioning and the MapReduce paradigm (Dean and Ghemawat, 2008; Banharnsakun, 2017). Data partitioning improves performance by executing clustering algorithms in data partitions in parallel and combining their results to produce global clusters (Dafir et al., 2020). In recent years, GPUs have gained increased attention because their massive parallel architecture can accelerate data intensive applications. Today, accelerated approaches combine multicore and GPUs present in modern laptops and desktop computers to build GPGPU applications. Some parallel traditional clustering algorithms based on data partitioning on GPGPUs include Kmeans and fuzzy c-Kmeans (Fakhi et al., 2017; Jamel and Akay, 2019), CUDA Kmeans (Giuroiu and keng Liao, 2015; Cuomo et al., 2019), clustering based on density G-DBSCAN (Andrade et al., 2013), and hierarchical clustering (Chen et al., 2017). A recent survey (Dafir et al., 2020) provides a discussion of parallel clustering algorithms and platforms.

For applications based on tractography datasets, some methods introduce parallelism using GPUs to improve the execution times for visualization of bundles and streamlines (Guevara et al., 2015; Combrisson et al., 2019), visualization of fused DTI/HARDI data (Prckovska et al., 2011), efficient tractography compression, storage, and visualization (Haehn et al., 2020), fiber segmentation (Ros et al., 2011; Labra et al., 2017), dMRI non-linear model fitting and probabilistic tractography calculation (Hernandez-Fernandez et al., 2019), geodesic fiber tracking (van Aart et al., 2011), and connectome pruning (Kumar et al., 2019).

In the context of fiber clustering, FFClust is a state-of-the-art fast method that builds high-quality clusters (Vázquez et al., 2020). The algorithm consists of four steps where it combines data parallelism on multicore architectures using local clustering, information aggregation, refinement, and graph representation to produce global clusters.

This work proposes a GPGPU parallel algorithm for FFClust. The parallel algorithm exploits multicore and GPU fine-grained parallelism. The proposed method provides a variation of the Kmeans++ algorithm, includes highly parallel patterns, memory optimization using constant, shared, and coalescing memory for high performance. The algorithms are implemented in C++, the thrust library, and the Compute Unified Device Architecture (CUDA) language. The experimental evaluation shows that our approach obtains clustering results with a quality that is similar to FFClust. In addition, it can attain an execution time of 3.5 s for about a million fibers, achieving a speedup of 11.5 times compared to FFClust. As far as we know, this is the first fiber clustering method that takes advantage of both multicore and GPU architectures, and it is the fastest streamline cluster algorithm in the research community, making it appealing for a variety of applications, including multi-subject clustering, WM bundle atlases, connectivity-based parcellation, and visualization tools.

## 2. Materials and Methods

### 2.1. Tractography Datasets

This study uses the ARCHI database (Schmitt et al., 2012) containing high-quality MRI data acquiered on a Tim Trio 3T MRI system with a 12-channel head coil (Siemens, Erlangen). The MRI protocol includes a T1-weighted image at 1 mm isotropic spatial resolution using an MPRAGE sequence, a *B*0 field map to correct artifacts, and a single-shell HARDI SS-EPI sequence with 60 optimized diffusion weighted directions, *b* = 1, 500 *s*/*mm*^2^ and isotropic spatial resolution of 1.7 mm. The HARDI dataset was corrected for artifacts produced by eddy currents, susceptibility effects, spikes, and noise. Then, the analytic q-ball diffusion model (Descoteaux et al., 2007) was calculated. A robust brain white matter propagation mask based on a T1-weighted segmentation was also calculated (Guevara et al., 2011a) and whole-brain regularized streamline deterministic tractography (Perrin et al., 2005) was computed on the diffusion-weighted (DW) space, based on the propagation mask, using a step of 0.2 mm and a maximum curvature angle of 30°. We used the BrainVISA / Connectomist-2.0 software to processed all data (Duclap et al., 2012). Resulting tractography datasets contain about one million fibers per subject (1,019,160 fibers on average). Also, we performed a post-processing step to resample all fibers using 21 equidistant 3D points. Therefore, each fiber consists of 21 3D points. Several previous approaches used the same representation (Guevara et al., 2012; Vázquez et al., 2020).

### 2.2. Background

#### 2.2.1. FFClust

**Figure 1:**
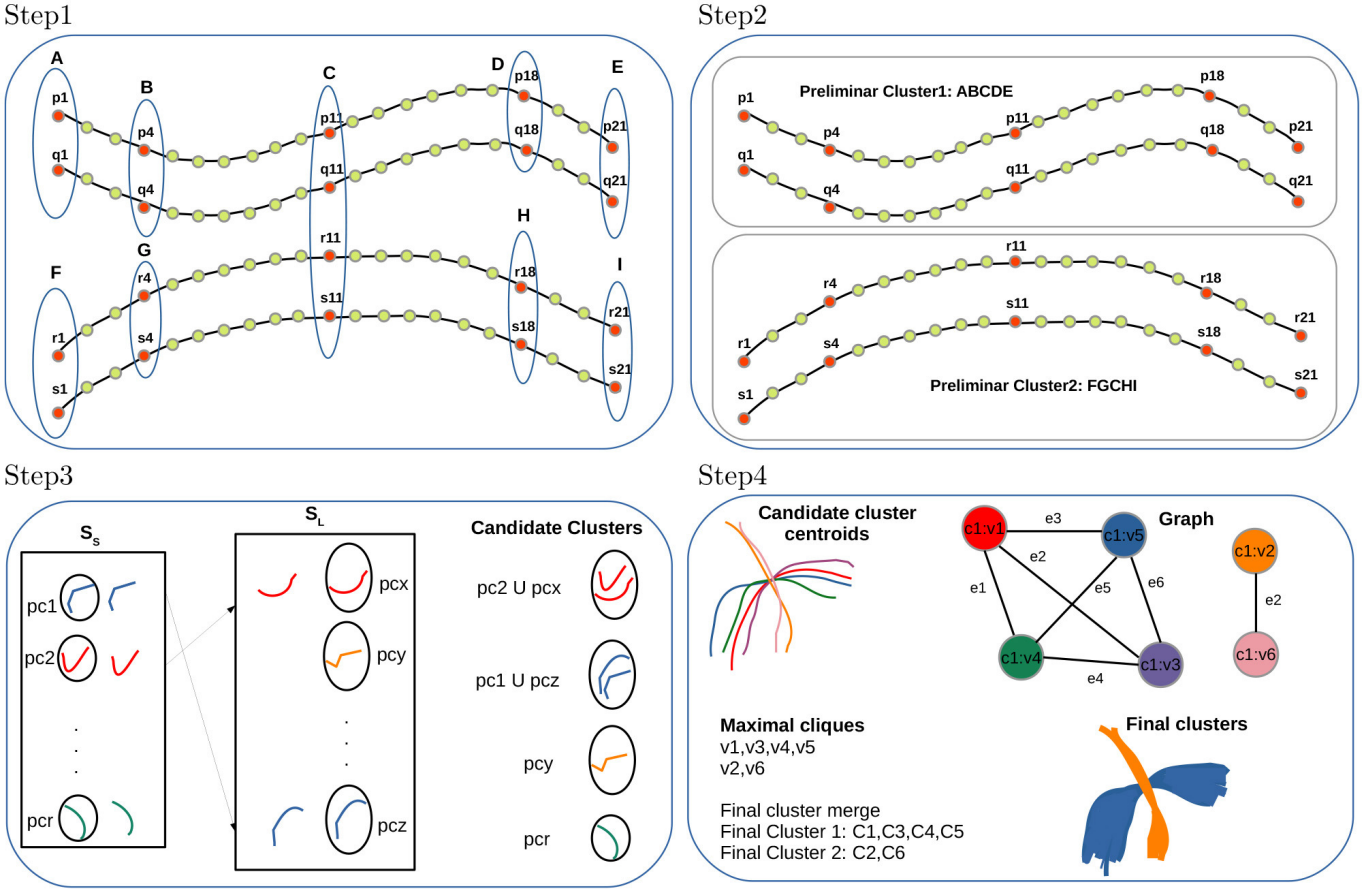
FFClust method with its four steps. **Step 1**: Building data point clusters, **Step 2**: Generating preliminary fiber clusters, **Step 3**: Reassignment of small to large preliminary clusters, **Step 4**: Building final clusters.

The FFClust algorithm consists of four steps described next and shown in Figure 1. The scheme uses the Euclidean distance expressed in *d*_*P*_ (Equation 1) for fiber 3D points, and Euclidean distance between fibers as given in Equations (2), (3), and (4). The *d*_*ME*_ distance is the minimum of the maximum Euclidean distance between the corresponding points of two fibers, considering fibers stored in direct (*d*_*E*_), and reverse or flipped (*d*_*EF*_) order in memory. Both storing orders are considered for a pair of fibers since for whole-brain tractography it is not possible to have a unique valid orientation for all fibers.

$$\begin{array}{r} d_{P}\left(a_{i},b_{i}\right)=||a_{i}-b_{i}|| \end{array}$$

(1) $$\begin{array}{r} =\sqrt{{\left(a_{i_{x}}-b_{i_{x}}\right)}^{2}+{\left(a_{i_{y}}-b_{i_{y}}\right)}^{2}+{\left(a_{i_{z}}-b_{i_{z}}\right)}^{2}} \end{array}$$

(2) $$\begin{array}{r} d_{E}\left(a,b\right)=max_{i\in 21}\left(d_{P}\left(a_{i},b_{i}\right)\right) \end{array}$$

(3) $$\begin{array}{r} d_{EF}\left(a,b\right)=d_{E}\left(a,b^{F}\right)=d_{E}\left(a^{F},b\right) \end{array}$$

(4) $$\begin{array}{r} d_{ME}\left(a,b\right)=min\left(d_{E}\left(a,b\right),d_{EF}\left(a,b\right)\right) \end{array}$$

*Step 1: Building data point clusters*. This step applies the Minibatch Kmeans (MK) (Sculley, 2010) on a subset of 3D fiber data points independently, using a different number of clusters depending on the position in the sequence of the 21 3D points in the fibers. The algorithm defines *K*_*pc*_ as the number of clusters used in the middle data point position and *K*_*po*_ as the number of clusters in the other selected data point positions. The value for *K*_*pc*_ is 200 and for *K*_*po*_ is 300; and the chosen data points positions are 1, 4, 11, 18, 21 (the two extreme points, the central points and two intermediate points). Therefore, the number of clusters chosen for the data point position 11 is 200, and the number of clusters for the point positions 1, 4, 18, 21 is 300. The algorithm used the Elbow method to obtain the number of clusters *K*_*pc*_ and *K*_*po*_ experimentally. At the end of this step, after applying MK on the five selected data points positions independently, all data points belonging to the specific positions are classified as belonging to a cluster identified by its membership. Figure 1 (Step 1) shows an example of cluster points for the selected positions 1, 4, 11, 18, 21 of fibers *p, q, r, s*, and the point cluster memberships obtained by MK are identified by labels *A, B, C, D, E, F, G, H, I*.

*Step 2: Generating preliminary fiber clusters*. This step builds preliminary fiber clusters using a dictionary data structure that groups all fibers sharing the same point cluster memberships obtained in Step 1. Figure 1 (Step 2) displays an example with two preliminary clusters. One is given by fibers *p, q* sharing the cluster point memberships *A, B, C, D, E* and the other contains fibers *r, s* sharing memberships *F, G, C, H, I*. The main caveat of Step 1 and Step 2 is that some close fibers might be classified incorrectly in different preliminary clusters because, in memory, some fibers reside in direct order and others in reverse order. The Step 3 of the algorithm addresses this problem by merging such preliminary clusters.

*Step 3: Reassignment of small to large preliminary clusters*. In this step, FFClust divides all preliminary clusters into two sets, a set with all clusters with six or more fibers (*S*_*L*_) and a set with small clusters containing all clusters with fewer than six fibers (*S*_*S*_). Then, the step defines a distance threshold *d*_*Rmax*_, computes cluster centroids using arithmetic mean of fiber points, and reassigns each small cluster to the closest large preliminary cluster if the distance *d*_*ME*_ between cluster centroids is less than *d*_*Rmax*_. The *d*_*ME*_ distance captures preliminary clusters consisting of close fibers that reside in memory with different orientation order. The value for the threshold *d*_*Rmax*_ is defined experimentally at 6 mm. At the end of the step, we obtain candidate clusters along with their centroids. Figure 1 (Step 3) displays an example of this reassignment, where the small preliminary cluster *pc1* is merged into the large preliminary cluster *pcz* forming a candidate cluster because the *d*_*ME*_ distance between their centroids is lower than the threshold *d*_*Rmax*_. Similarly, preliminary cluster *pc2* is merged into *pcx*. Not merged preliminary clusters containing more than two fibers such as *pcr* and *pcy* also become candidate clusters. At the end of this step, preliminary clusters with one or two fibers are discarded as noise.

*Step 4: Building final clusters*. The last step performs the final refinement process and produces the merging of the candidate fiber clusters obtained in Step 3. This step first builds groups defined by the membership of the middle point of fibers obtained in Step 1. Then a graph representation is defined for each group. In the graph representation, each centroid of a candidate cluster is represented as a vertex, and an edge is defined if two vertices have a maximum distance *d*_*ME*_ below the threshold *d*_*Mmax*_. This threshold is set at 6 mm. After building the graph, the algorithm finds all maximal cliques, sorts them by decreasing size, and merges candidate clusters belonging to the same maximal clique. Note that candidate clusters are merged according to the first processed maximal clique. Figure 1 (Step 4) depicts a high-level overview of the step, including an example of candidate cluster centroids, graph representation, maximal cliques, and merge to produce the final clusters. In the example, there is one group with six candidate cluster centroids represented by a graph with six vertices (*vi*, *i* ∈ [1, 6]) and seven edges (*ej*, *j* ∈ [1, 7]). There are two maximal cliques in the graph; one has the vertices *v*1, *v*3, *v*4, *v*5, and the other has vertices *v*2, *v*6. Then, candidate clusters *C*1, *C*3, *C*4, *C*5 are merged into one final cluster (blue cluster), and candidate clusters *C*2, *C*6 into another final cluster (orange cluster).

#### 2.2.2. Kmeans Algorithm

This work uses the Kmeans method instead of the MK algorithm in the Step 1 of FFClust. Kmeans is a well-known unsupervised partition-based clustering algorithm that classifies input data points into *K* partitions based on a distance metric. The algorithm is iterative and consists of the following steps.

1. Choose *K* random centroids from data points
2. Repeat until convergence or number of iterations
  - Assign each data point to the cluster with minimum distance to centroid
  - Compute centroids based on partitions produced in previous step

The Kmeans algorithm has two main phases described in points 2.a and 2.b. The 2.a phase, where each data point finds its closest centroid, has a sequential time complexity of *O*(*NKd*), where *N* is the number of data points, *K* is the number of clusters, and *d* the data point dimension. The 2.b phase of the algorithm, where each cluster recomputes its centroid, has a sequential time complexity of *O*(*N* + *K*). Note that typically *N* >> *K*, then the time complexity ratio between phase 2.a and 2.b is *O*(*Kd*), which means that phase 2.a grows faster than 2.b and therefore parallelizing phase 2.a has more impact on the speedup. Phase 2.a provides high parallelism since there is no data dependency, where each processor can compute the distance of a data point to the *K* cluster centroids and associate the data point to the cluster id with the minimum distance. Thus, this phase is bounded by the number of available parallel processors. The parallel time complexity of this phase is given by *O*(*NKd*/*n*_*p*_), where *n*_*p*_ is the number of processors. Observe that the ratio between the parallel 2.a phase and sequential 2.b is *O*(*NKd*/*n*_*p*_(*N* + *K*)), and if *N* >> *K*, which is the usual case, it is simplified to *O*(*Kd*/*n*_*p*_). Then, it is crucial to accelerate phase 2.a. As observed, the parallelism is limited by the number of available processors. Given this analysis, we define a parallel algorithm using GPU parallelism since it allows to exploit more parallelism using more threads than using a multi-core CPU.

Kmeans++ is a variation of Kmeans that improves the quality of the clusters. The algorithm is identical to Kmeans, except for the initialization of the *K* centroids. Kmeans++ selects the *K* initial centroids that are farther apart, starting from a random point. This selection intends to avoid the incorrect division of clusters. Using Kmeans++ cluster centroid initialization also exploits parallelism in the GPU as described in section 2.3.

### 2.3. Parallel Algorithms for FFClust

**Figure 2:**
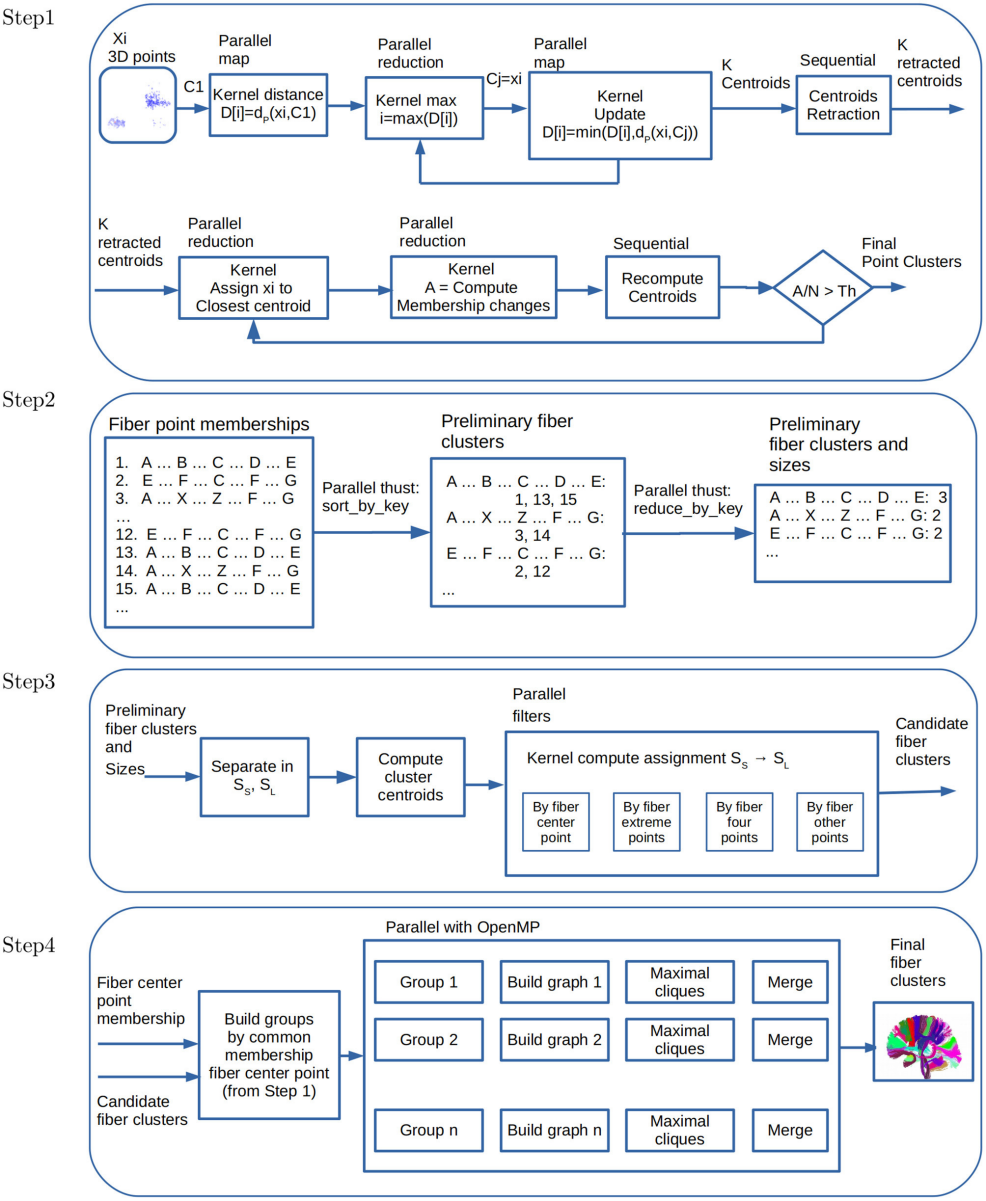
Data flow and parallelism in GPU kernels. **Step 1**: Parallel point clustering, using Kmeans with point retraction computation with CUDA kernels based on parallel map and maximum and minimum reduction operations. **Step 2**: Building preliminary fiber clusters using parallel *sort_by_key* and *reduce_by_key* operations from *thrust* library. **Step 3**: Building candidate fiber clusters by reassignment of small to large preliminary clusters using point filters with CUDA kernels. **Step 4**: Building final fiber clusters with graph representation using OpenMP thread parallelism. Candidate clusters are separated in groups, where each group shares the fiber center point membership of *Step 1*, and then each thread creates and process a graph to produce the final clusters.

This section describes the parallel algorithms performed in each of the steps of FFClust. Figure 2 shows the data flow of our parallelization scheme.

#### 2.3.1. Parallel Algorithms for Step 1 of FFClust

As mentioned in the previous section, Step 1 of FFClust applies the MK algorithm, which uses random samples to reduce the execution time. Instead of MK, this work studies algorithms based on Kmeans and Kmeans++ using all the data points. In particular, this study provides parallel algorithms for Kmeans++ based on the CUDA Kmeans (Giuroiu and keng Liao, 2015). As this step has the longest execution time, our parallel approach considers different alternatives. The first alternative uses a parallel algorithm for Kmeans, the second uses Kmeans++, and the third alternative proposes a variation of Kmeans++. All these implementations use coalescing memory access patterns, constant, and shared memory in the GPU to improve performance.

#### 2.3.2. Step 1.a. Building Point Clustering Using Parallel Kmeans

For this alternative, we use the basic algorithm of parallel Kmeans proposed by Wei-keng Liao and Serban Giuroiu (Giuroiu and keng Liao, 2015). We applied it on the same fiber point positions that FFClust uses in its Step 1, that is, the positions {1, 4, 11, 18, 21} with *K*_*pc*_ = 200 and *K*_*po*_ = 300.

#### 2.3.3. Step 1.b. Building Point Clustering Using Parallel Kmeans++

This step describes the Kmeans++ algorithm, aiming to improve the selection of initial centroids used by the basic Kmeans algorithm. The basic Kmeans algorithm chooses initial centroids at random. The problem with this initialization is that final clusters might produce clusters incorrectly partitioned. On the other hand, Kmeans++ aims to select the initial centroids from data points that are far away from one another. This scheme aims to select data points from different clusters as initial centroids, which aids in building better clusters than Kmeans.

Algorithm 1 shows the main algorithm for implementing Kmeans++. For a selected position in all fibers in the tractography dataset *T*, the algorithm first defines an initial centroid *C*_1_ by choosing a data point at random. Then, it computes the array *D* with the Euclidean distance for every data point *x*_*i*_ ∈ *T* to the centroid *C*_1_. Note that here the Euclidean distance is the distance between fiber 3D points (*d*_*P*_ in Equation 1). Next, the algorithm defines as the next centroid a data point *x*_*i*_, which is the farthest away (maximum distance) point to all previous centroids (lines 6 and 7 in the algorithm). The algorithm maintains in *D* the minimum distance of every point in *T* to all previously obtained centroids; that is, for every point *x*_*i*_, it stores the distance to the closest previous chosen centroid (lines 8 to 10 in the algorithm).

**Algorithm 1:** **Kmeans++ algorithm**.

**Require**: *T*, tractography dataset on fiber data point
**Require**: *k*, number of clusters, *d*_*P*_
1: *C*_1_ ← *getRandomPoint*(*T*)
2: **for** *i* = 1 to |*T*| **do** ⊳ with parallel MAP
3: D[i] = *d*_*P*_(*x*_*i*_, *C*_1_)
4: **end for**
5: **for** *j* = 2 to *k* **do**
6: *i* = max{*D*[*j*]} ⊳ get position of maximum value in *D* with parallel max Reduction operation
7: *C*_*j*_ = *x*_*i*_
8: **for** *i* = 1 to |*T*| **do** ⊳ Update *D* with parallel MAP
9: D[i] = min{*D*[*i*], *d*_*P*_(*x*_*i*_, *C*_*s*_)}, *x*_*i*_ ∈ *T, s* ∈ [1, *j*]
10: **end for**
11: **end for**
12: return *C*_*s*_, *s* ∈ [1, *k*]

The algorithm just described has fine-grained data parallelism. First, the distance array *D* can be computed using a Map parallel operation since it applies the distance function to every point *x*_*i*_ ∈ *T*, and an independent thread for each point can perform this operation. This operation takes *O*(1) parallel time. Next, the nested loop in the algorithm obtains the position of the farthest point to centroids, and it can be performed by using a maximum parallel Reduction operation, which is *O*(*log*(*N*)). Then, the algorithm performs a parallel Map, keeping the minimum distance for each data point in the *D* array. The algorithm obtains the next initial centroid computing the data point at maximum distance in *D*.

#### 2.3.4. Step 1.c. Centroid Retraction: A Variation of Kmeans++ to Avoid Outliers

As mentioned, Algorithm 1 aims to define data points as initial centroids that are far from each other using Kmeans++. However, this algorithm might choose as initial centroids data points that are outliers, that is, points that are far from any other point. To avoid this problem, we define an operation to move initial centroids toward the mean of the centroids. Note that these initial centroids will probably be different from actual data points. However, this is the way all centroids are updated in the iterative process of Kmeans. We call this processing a retraction. This computation is somehow similar to what the clustering algorithm CURE (Guha et al., 2001) does to choose cluster representatives.

The operation for computing the point retraction *q*_*i*_ is given in Equation (5),

(5) $$\begin{array}{r} q_{i}=C_{i}\left(1-r\right)+C_{m}\times r \end{array}$$

where *r* is the retraction rate defined as a value in the range [0..1] and $C_{m}=\frac{\underset{i\in \left[1,k\right]}{\sum }C_{i}}{K}$. As this operation is performed once on the initial centroids per selected point, its computation time is linear with the number of clusters, *K*.

**Figure 3:**
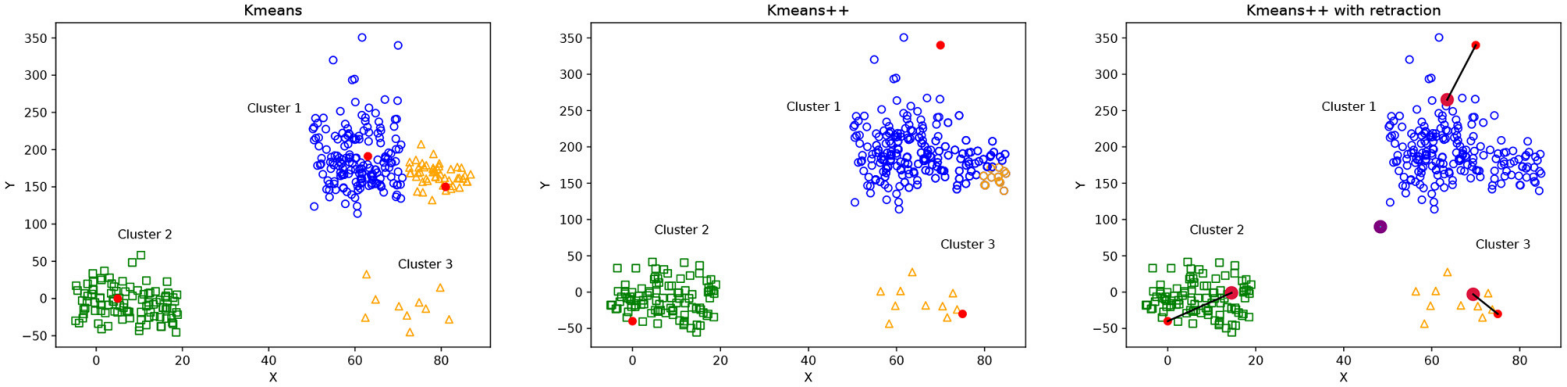
Illustration of possible cluster formation using Kmeans, Kmeans++ and Kmeans with point retraction. Initial centroid points are shown in red. Figure on the **(left)** shows that random centroids for the blue and orange clusters are too close, producing an incorrect cluster formation. The figure in the **(middle)** shows Kmeans++ centroid initialization, where the initial centroid for the blue cluster is far away from any other point, producing a wrong classification of some points which are assigned to the orange cluster. Figure on the **(right)** shows Kmeans++ with point retraction which defines initial centroids that provide a better cluster formation than Kmeans++.

Figure 3 illustrates the differences between the selection of initial centroids of Kmeans, Kmeans++ and Kmeans++ with point retraction. The Kmeans case (Figure 3, left) shows the potential problem of choosing random centroids, where the blue and orange clusters seem incorrectly separated because their corresponding initial centroids were probably too close. Kmeans++ (Figure 3, middle) can improve the Kmeans clustering, but it still can incorrectly separate the blue cluster because its initial centroid was too far away from all the cluster points. The Kmeans++ with point retraction can reduce the effect of an outlier centroid as shown in Figure 3 on the right.

Figure 2 (Step 1) displays the data flow and parallel kernels for computing Kmeans++ with retraction of initial centroids and the kernels used for data partitioning to compute the final fiber point clusters. Note that this step is performed for each of the five points selected positions 1, 4, 11, 18, 21 of fibers, resulting in fiber point clusters for each position.

#### 2.3.5. Parallel Algorithm for Step 2 of FFClust

In Step 1, as a result of computing Kmeans, we stored cluster fiber point memberships in a matrix of *N* rows and *p* columns, where *N* is the number of fibers and *p* = 5 the number of fiber points. Each column contains the cluster membership of each fiber point position. Consequently, each row contains the cluster memberships of each fiber point. This representation helps to process Step 2 and 3 of FFClust in the GPGPU implementation. In Step 2 we use this information to define a dictionary data structure where each row in the matrix is a key representing a fiber cluster point memberships, and the value is the fiber id. We use the parallel thrust library (nVIDIA Developer, 2020) primitives to compute this step. The first primitive is **sort_by_key()**, which receives as input an array of keys, and an array of values. It outputs the sorted keys with corresponding values, respectively. This output is precisely the FFClust definition of preliminary clusters, where each preliminary cluster is identified by a unique key, containing a collection of fiber ids. Figure 2 (Step 2) displays the operation of **sort_by_key()** using an example. In the example, there are three fiber groups identified with their corresponding fiber cluster point memberships {*A, B, C, D, E*}, {*A, X, Z, F, G*}, and {*E, F, C, F, H*}. The first group contains fibers {1, 3, 15}, the second contains fibers {3, 14} and the third contains fibers {2, 12}. In addition, we use the thrust **reduce_by_key()** primitive. This operation receives as input the sorted membership keys, and an array of values. It outputs the unique keys (i.e., memberships), and an array with the number of values (i.e., the number of fibers) that have the same key. In the example of Figure 2 (Step 2), the number of fibers in each of the groups are 3, 2, and 2, respectively. Step 3 uses as input the preliminary fiber clusters with their corresponding sizes.

Note that the **sort_by_key()** and **reduce_by_key()** Thrust parallel primitives implement the MapReduce pattern, which boosts programmer productivity and provide high performance as stated in Bell and Hoberock (2012).

#### 2.3.6. Parallel Algorithm for Step 3 of FFClust

In this step small preliminary clusters are reassigned to large clusters. We used the preliminary clusters with their corresponding sizes computed in Step 2. Based on the number of fibers, the preliminary clusters are classified into two sets: *S*_*S*_: Small clusters, clusters with fewer than six fibers, and *S*_*L*_: Large clusters, clusters with six or more fibers.

A centroid for each cluster is computed in parallel in a CUDA thread to perform the cluster reassignment. The reassignment using the centroids of preliminary clusters uses a pipeline of filters in parallel. First, it separates clusters by centroids that are close to each other based on the middle fiber data point. Each group is separated by sub-groups of fibers containing external fiber points close to each other. Next, the separation is done by four points, and finally with the other fiber data points.

Figure 2 (Step 3) displays the data flow for the parallel operations performed in this step.

#### 2.3.7. Parallel Algorithm for Step 4 of FFClust

This step uses a graph model, representing candidate clusters as vertices and defining edges based on a threshold of the maximum Euclidean distance of pairwise centroids. To improve performance, FFClust first groups centroids based on the membership of the middle point of fibers, and then over each group, it applies the graph model. Next, for each graph, the algorithm enumerates all maximal cliques, sorts them by size, and merges all clusters represented by the vertices in the same maximal clique. The FFClust implementation uses *networkx* python package to perform all graph operations. In this work, we follow the same ideas, but instead of using python *networkx*, we use C++ and the Eppstein et al. algorithm (Eppstein et al., 2013) for listing maximal cliques, which is very efficient for sparse graphs. Because this is a refinement step, it is expected that these graphs are likely to be sparse, then using an algorithm for listing maximal cliques on sparse graphs is expected to be more efficient. We use OpenMP parallelism instead of parallelism on the GPU in this step because the size and number of maximal cliques are rather small, and moving data to and from the device reduces the performance. Figure 2 (Step 4) displays the data flow for the used parallel operations, where each group and graph processing is in parallel.

## 3. Results

This section presents an experimental evaluation of the proposed method. First, we provide the main statistics of the results, including the coverage of fibers in final clusters, the histograms of the number of fibers in clusters, the intra-cluster and inter-cluster distances. Second, we present a visual inspection of the final clusters obtained by the methods. Next, we discuss the quality of cluster results using the Davies-Bouldin index (DB index) (Xu and Tian, 2015). Then, we analyze the performance by comparing execution times, speedup, and scalability of our parallel alternatives compared to FFClust. Finally, we compare the clustering results of FFClust and our best alternative for the segmentation of long bundles application. In all experiments, we used 10 tractography datasets representing 10 different subjects containing about one million fibers.

### 3.1. Main Statistics of Final Clusters

This section presents the main statistics obtained in this study. We use different names according to the alternatives defined in Step 1 to distinguish each of the parallel schemes. We denote the alternative that uses random initialization of centroids in Step 1 as CkFFC. The alternative with Kmeans++ initialization as CkpFFC, and CeFFC for the alternative of Kmeans++ with point retraction. CeFFC uses point retraction of 5%, which we obtained experimentally. In addition, we define an alternative that includes a post-processing step that removes all final clusters with intra-cluster distances over 70 mm. We called this alternative CefFFC.

**Figure 4:**
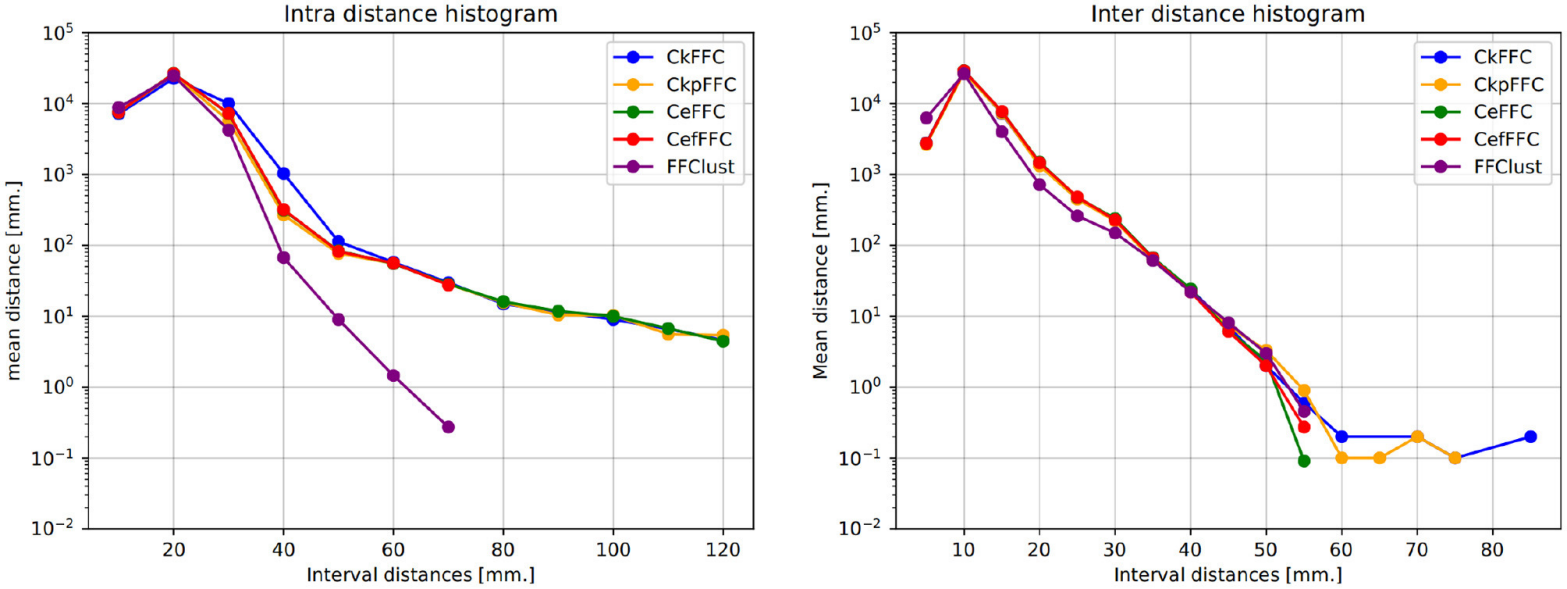
Intra-cluster and Inter-cluster distance histograms of final clusters. Intra-cluster distance shows that the parallel alternatives achieve less compact clusters than FFClust. Inter-cluster distance shows that parallel alternatives have fewer clusters that are closer to each other than FFClust.

Typically, clustering algorithms aim to obtain compact clusters, clusters with small intra-cluter distances; and well-separated clusters, clusters with large inter-cluster distances. Figure 4 (left) displays the histogram of the intra-distance of final clusters, and Figure 4 (right) the histogram of the inter-cluster distance. As observed, our alternatives obtain about 60 clusters with intra-cluster distance over 70 mm, whereas FFClust does not. However, the parallel alternatives obtain about five thousand clusters fewer than FFClust with inter-cluster distance less than 5 mm. This means that more clusters in FFClust are closer among one another than in the parallel methods.

**Figure 5:**
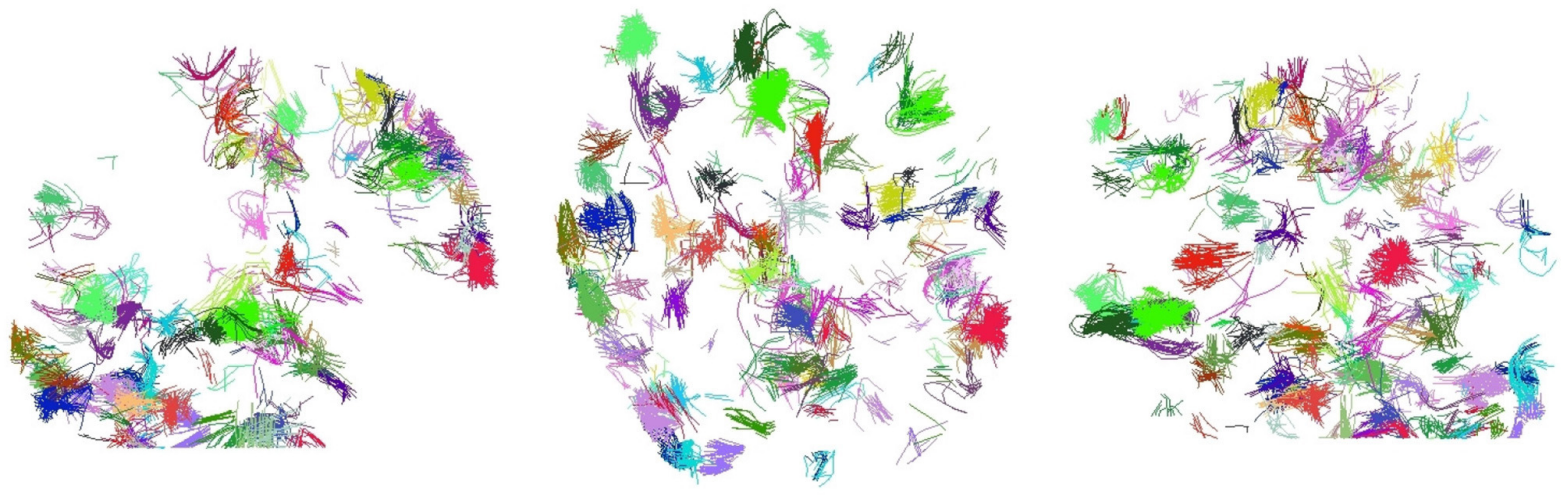
Final clusters with intra-cluster distance over 70 mm in CeFFC for a subject. With coronal, axial, and sagittal views. All these clusters are well formed, but contain short fibers that seem to be noise.

We explore the final clusters with intra-distance over 70 mm obtained in our alternatives. Figure 5 displays them. We observe that all of them are clusters consisting of short fibers. As observe, all appear to be noise. Since they do not provide relevant information, we believe that it is correct to eliminate those clusters after inspection.

**Figure 6:**
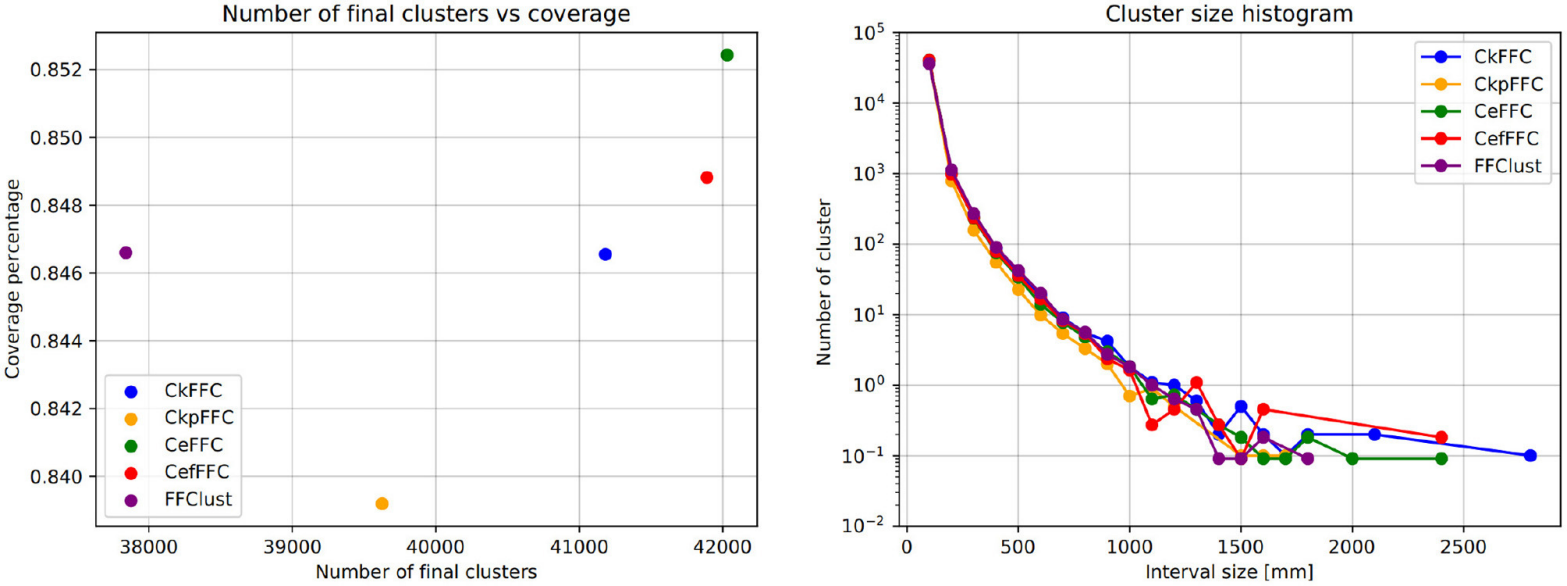
Coverage and size histogram of final clusters. The CeFFC alternative obtains the highest number of clusters and coverage of fibers. It also contains a few bigger clusters than FFClust.

Figure 6 (left) shows the coverage of fibers in all final clusters measured as a percentage of the total number of fibers. Figure 6 (right) displays the histogram of the cluster sizes. These figures show that the parallel implementations provide a similar fiber coverage to FFClust, between 83.8 and 85.2%, but they differ in the number of final clusters. The CkFFC obtains about 3200 more clusters than FFClust, which is about 8.4% more clusters. CkpFFC provides 1500 more clusters (3.4%), and CeFFC provides about 4, 100 more (10.8%). The cluster size histogram in Figure 6 (right) shows that the cluster sizes are similar, although the parallel alternatives find about two larger clusters than FFClust.

### 3.2. Cluster Result Visualization

**Figure 7:**
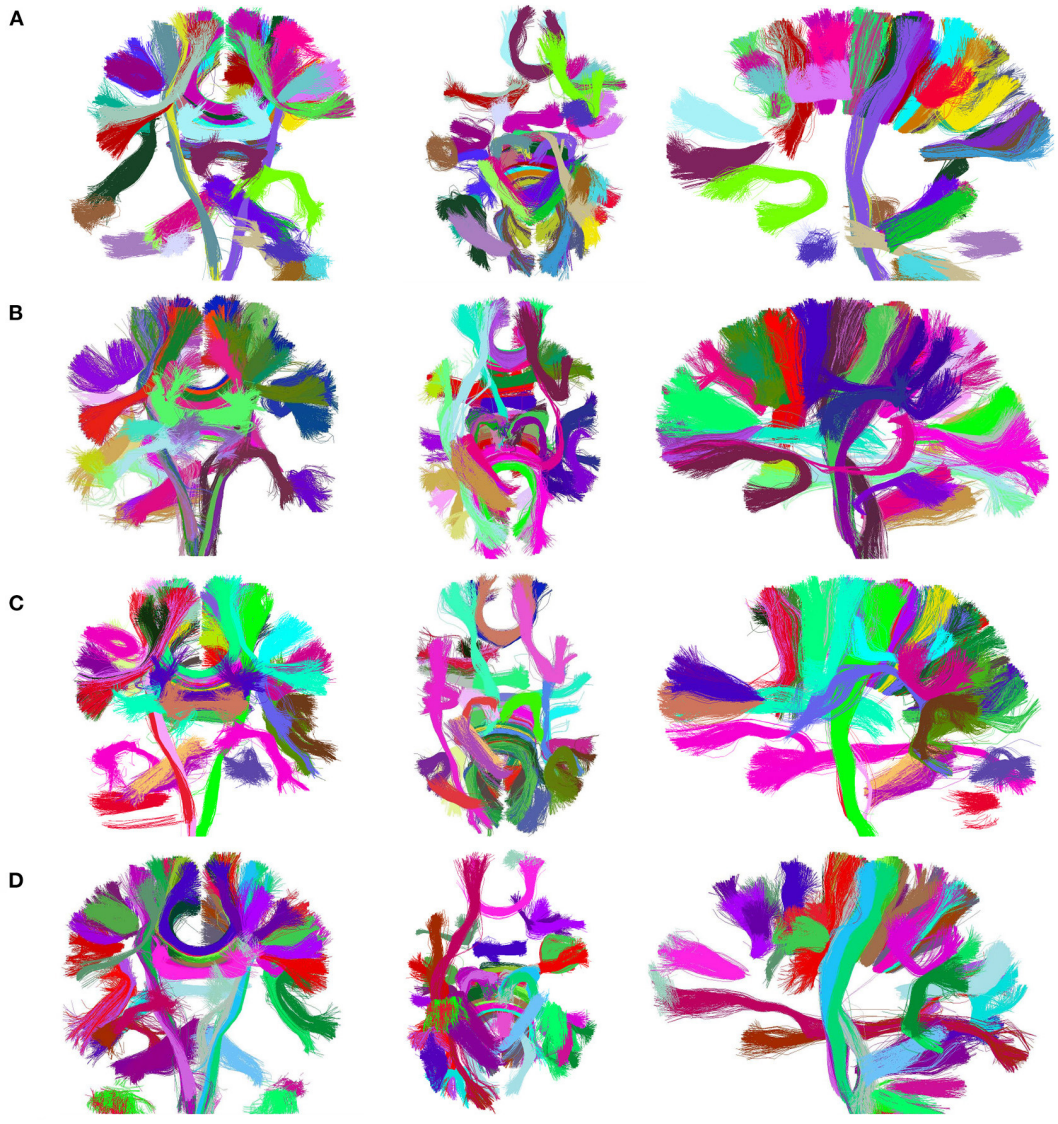
Visualization of 50 largest final clusters for a subject, for FFClust and the three parallel methods (CeFFC, CkpFFC, and CkFFC). The CeFFC alternative obtains well formed clusters with a higher brain area coverage than FFClust for this group of clusters. FFClust shows more compact clusters than the parallel alternatives. **(A)** FFClust. **(B)** CeFFC. **(C)** CkpFFC. **(D)** CkFFC.

Figure 7 presents the visualization of the 50 largest final clusters for all the alternatives. We omitted CefFFC because the 50 largest clusters are the same as the CeFFC alternative. The visualization includes the coronal, axial, and sagittal views for each method. In general, the results are quite similar between the methods. As observed, all methods provide well-formed clusters covering the main whole brain structure. The 50 bigger clusters correspond to portions of the corpus callosum, corticospinal tract, uncinate fasciculus and some short connections. As we used an arbitrary cluster size threshold, some parts of the brain my not be covered by the selected clusters. FFClust seems to produce more compact clusters, the alternative with Kmeans++ with point retraction (CeFFC) appears to achieve a better coverage of the brain, while the alternative with Kmeans with random initialization of centroids (CkFFC) presents more portions of the brain with fewer clusters. To complement this exploratory analysis, section 3.6 provides a comparison of FFClust and CeFFC methods based on the segmentation of deep white matter bundles.

### 3.3. Quality of Clusters

This study measures the quality of final clusters by using the Davies-Bouldin index (DB index, Xu and Tian, 2015). The DB-index is a measure defined as the average similarity of each cluster with its most similar cluster. The similarity is the ratio of intra-cluster distances to inter-cluster distances (Equation 6). Thus, a higher-quality clustering is achieved when elements belonging in clusters are compact or less disperse, and different clusters are farther apart among them.

Since our goal is to produce a high-quality clustering algorithm close to FFClust, we define a normalized DB index (*DB*_*n*_), which divides the DB index obtained by an alternative algorithm, by the DB index of FFClust.

The normalized DB-index is presented in Equation (7). The closest value of *DB*_*n*_ index to 1 means that the method is the closest in *DB* to FFClust. A value greater than one means that the alternative method has a better DB index than FFClust. In addition, Equation (8) presents the variance, which we used to evaluate how DB index varies between execution trials.

(6) $$\begin{array}{r} DB=\frac{1}{n}\sum_{i=1}^{n}\underset{i\neq j}{\max}\left(\frac{\alpha_{i}+\alpha_{j}}{d\left(c_{i},c_{j}\right)}\right) \end{array}$$

(7) $$\begin{array}{r} DB_{n}=\frac{DB_{ffclust}}{DB_{m}} \end{array}$$

(8) $$\begin{array}{r} Var\left(X\right)=\frac{\overset{n}{\underset{1}{\sum }}{\left(x_{i}-\mu \right)}^{2}}{n} \end{array}$$

**Figure 8:**
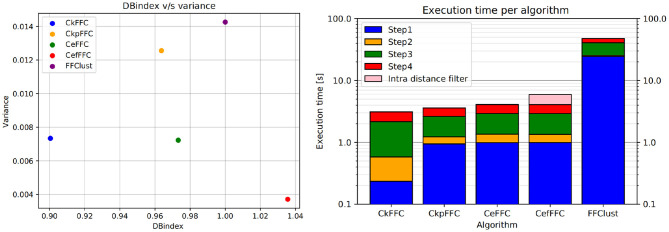
Normalized DB-Index on the left and Execution times in log scale on the right. The CeFFC alternative achieves a *DB* almost as good as FFClust. The CeFFC alternative, which filters out clusters with intra-distance over 70 mm, achieves a slightly better *DB* than FFClust. All parallel alternatives improve execution times of FFClust in an order of magnitude. Note that the lowest execution time value in Y axis is 0.1 s.

Figure 8 (left) shows the *DB*_*n*_ index vs. the variance obtained for the analyzed methods using the 10 tractography subjects. As seen in the figure, FFClust has the highest variance of 0.014. In addition, CkpFFC and CeFFC provide the closest alternatives to FFClust, but CeFFC is the closest to FFClust. The CefFFC alternative provides a *DB*_*n*_ of 1.038, which means achieving a DB index slightly better than FFClust.

### 3.4. Performance Results

We evaluate the performance in terms of execution time and speedup. We implemented the parallel algorithms using C++, thrust library, OpenMP, CUDA 10 toolkit with O3 compiler optimization. All experiments were executed in a i5-9600K 6C12H CPU model, which has 6 cores working at 3.7GHz; 32 GB of RAM and a Quadro P620 Nvidia GPU.

Figure 8 (right) displays the arithmetic mean of the execution time achieved in each step of the algorithms for 10 tractography datasets with about a million fibers each. As observed, Step 1 consumes the largest execution time in FFClust, whereas using fine-grain parallelism in all the presented alternatives improves the execution time of this step in more than one order of magnitude. This figure also shows that all parallel alternatives achieved a similar total execution time between 2.5 and 4 s, while FFClust consumes about 40 s. The CefFFC alternative filters out all final clusters produced by CeFFC that have intra-cluster distance over 70 mm. This post-processing is also parallelized and increases the execution time by about 2 s (shown in pink in Figure 8, right), achieving a total of 6 s.

**Figure 9:**
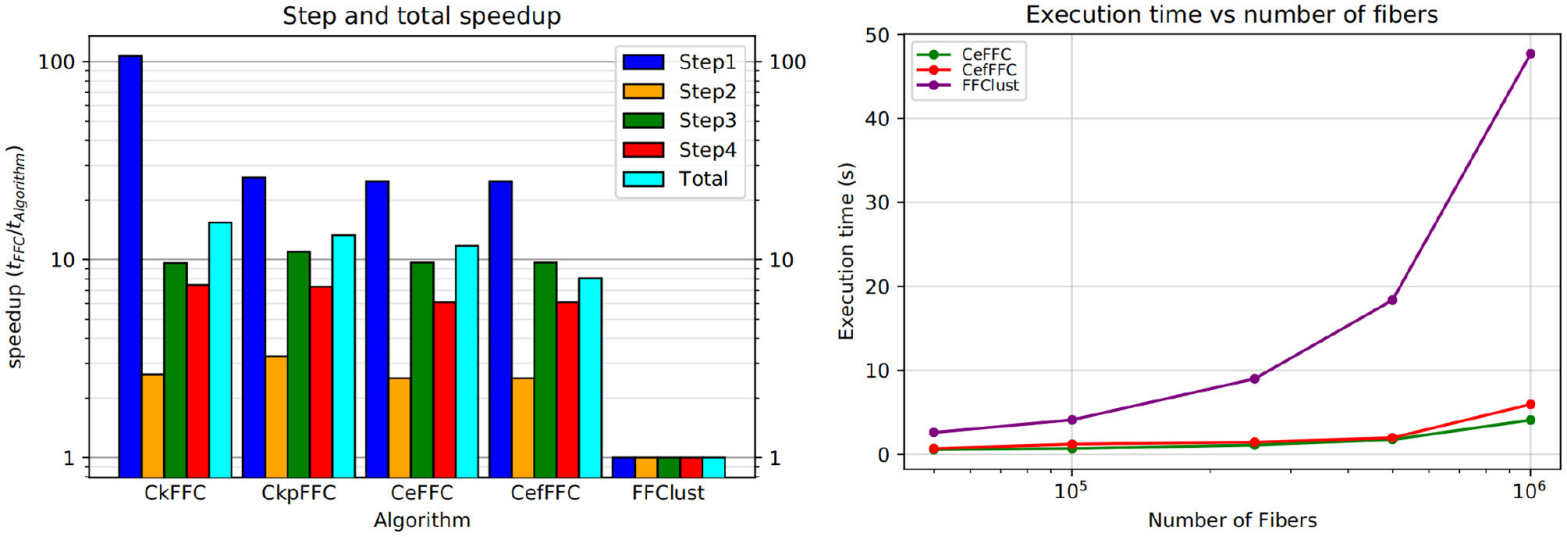
Speedup and scalability of our parallel alternatives of FFClust. All parallel alternatives obtain a speedup greater than two in all the steps of FFClust, where the highest speedup is achieved in the Step 1 and the lowest in Step 2. The scalability test on the right figure shows that the execution time of the parallel alternatives is rather stable with the number of fibers, whereas the execution time of FFClust increases with the number of fibers.

Figure 9 (left) shows the speedup achieved for the parallel alternatives in each of the steps of the algorithm. As seen in the figure, the most significant gain in speedup is in the first step, where the algorithms CkFFC provides a speedup around 80, whereas in CeFFC and CkpFFC is about 20. The second best speedup is in Step 3, where it is over 10, and Step 4 follows with a speedup between 8 and 9. The Step 2 of the algorithm has the lowest speedup, which is about two. Overall, considering all steps, the parallel algorithms achieved a speedup of 11.5 over FFClust.

Figure 9 (right) displays a scalability test comparison between FFClust and CeFFC in the *Step 1* of FFClust. The figure shows that increasing the number of fibers does not increases much the execution time.

### 3.5. Performance Discussion

Table 1 displays a comparison summary between FFClust and the best GPGPU implementation, CeFFC. For each of the steps, it shows the programming languages, libraries, computing platforms, and the execution times in seconds required. In addition, the last column of the table provides the speedup achieved by CeFFC in each Step. As observed, FFClust exploits CPU parallelism in all the Steps, where Step 1, 2 and 4 uses python Multiprocessing package for process-based parallelism. In Step 3, FFClust uses parallelism using C with OpenMP (Vázquez et al., 2019). The GPGPU implementation uses C++ and CUDA in Step 1, CUDA Thrust library in Step 2 and 3, and C++ and OpenMP in Step 4. As seen in the last column of Table 1, the GPGPU implementation achieves acceleration in all the FFClust steps.

**Table 1:** Comparison summary by steps between FFClust and our best parallel implementation CeFFC using a tractography of a million fibers.

**Algorithm**	**FFClust**			**CeFFC**			
**Steps**	**Prog. languages**	**Libraries, parallelism**	**Time (s)**	**Prog. languages**	**Libraries, parallelism**	**Time (s)**	**Speedup**
Step 1	Python	Multiprocessing, MiniBatchKMeans	20.10	C++	CUDA Kmeans*	0.99	20.30
Step 2	Python	Multiprocessing	0.88	C++	Thrust	0.40	2.20
Step 3	Python, C	OpenMP	12.82	C++	CUDA	1.25	10.25
Step 4	Python	Multiprocessing, networkx	7.41	C++	OpenMP, Eppstein	0.92	8.05
Total exec. time (s)			41.21			3.56	11.57

Kmeans* denotes Kmeans++ with point retraction.

In Step 1, the FFClust algorithm uses **MiniBachKMeans** and **Multiprocessing** python packages, whereas the GPGPU alternative implements in C++ and CUDA the Kmeans++ with point retraction for centroid initialization. Moreover, since MiniBatchKMeans uses sampling, it is faster than the other Kmeans implementations in python. We also implemented sampling with Kmeans++, but we obtained lower quality results and the execution time saving was not significant and therefore not shown in this study. Table 1 shows that we achieve a speedup of 20.30 in this Step.

In Step 2, FFClust uses the Multiprocessing python package to parallelize the construction of a dictionary data structure to compute preliminary fiber clusters. In contrast, the GPGPU implementation uses the parallel thrust primitive **sort_by_key()** to compute the preliminary fiber clusters and the **reduce_by_key()** primitive to compute the size of preliminary fiber clusters required as input in the Step 3. Table 1 shows we achieve a speedup of 2.20 in this Step.

In Step 3, FFClust defines a C and OpenMP parallel implementation (Vázquez et al., 2019), which is called from the python implementation. Our GPGPU implementation uses the GPU to exploit more parallelism since more work is performed to assign small preliminary clusters to large ones. As observed in Table 1, the execution time of Step 3 in FFClust is 12.82 s, whereas in Step 3 of the GPGPU implementation is 1.25 s achieving a speedup of 10.25. Since we use the same algorithm and C/C++ as the programming language, the gain in the GPGPU implementation comes mostly from using more processors.

Step 4 of the FFClust algorithm uses python *networkx* package, which is based on the Tomita algorithm (Tomita et al., 2006) for enumerating maximal cliques^1^. As discussed in Eppstein et al. (2013) the Tomita algorithm is an implementation of the Bron and Kerbosch' algorithm (Bron and Kerbosch, 1973), using a heuristic based on a pivoting strategy described by Cazals and Karande (2008). Eppstein et al. (2013) show, experimentally, that their algorithm is between 10 and 160 times faster than Tomita's algorithm for sparse graphs. In Step 4 of the GPGPU implementation, we use the Eppstein algorithm to enumerate maximal cliques. Given that this algorithm does not exhibit evident SIMD parallelism, we did not define a GPU parallel algorithm for this step. However, using OpenMP parallelism and the Eppstein algorithm in this step provide a speedup of 8.05 as shown in Table 1.

Finally, Table 1 displays that the overall speedup of the GPGPU implementation of FFClust is 11.57.

### 3.6. Segmentation of Deep White Matter Bundles

**Figure 10:**
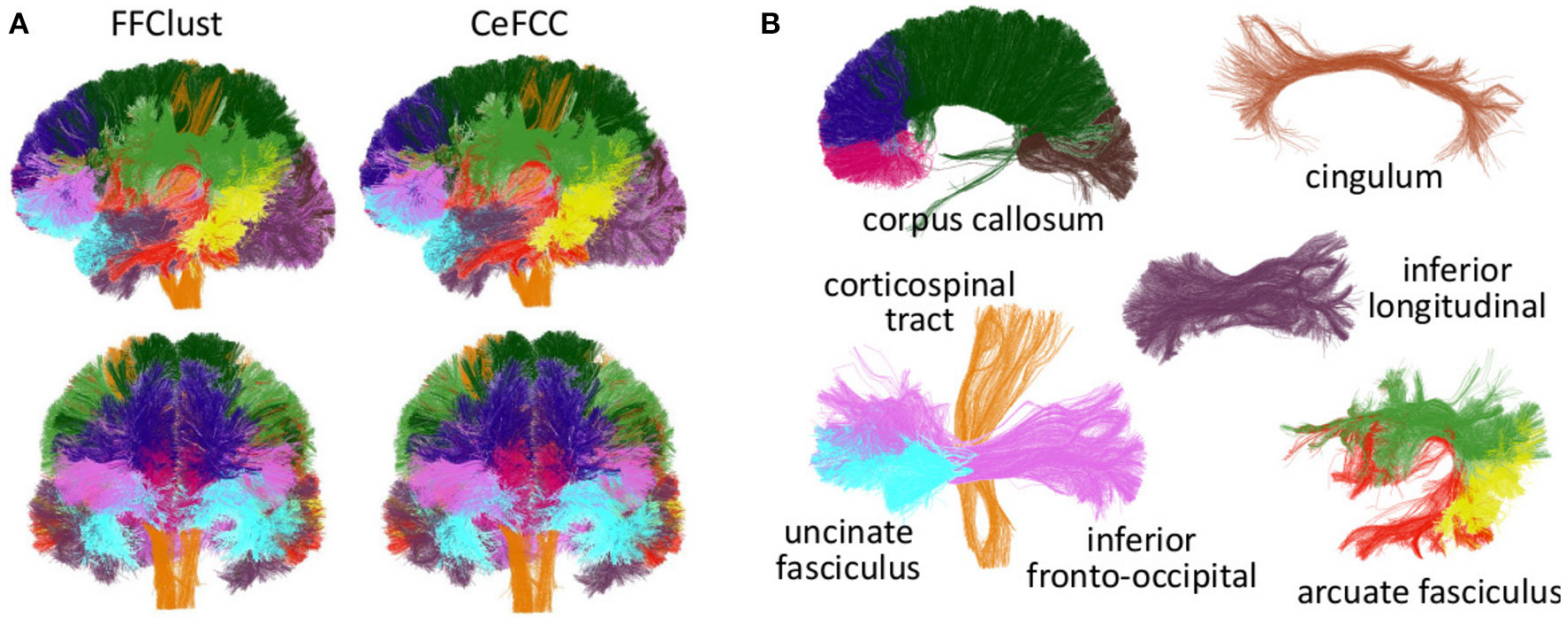
Segmented bundles for FFClust and CeFFC algorithms (one subject). **(A)** All the segmented bundles. First row: left sagittal view, second row: coronal view. **(B)** The segmented bundles on separated displays for CeFFC algorithm (left sagittal view). Corpus Callosum: rostrum (magenta), genu (blue), body (dark green) and splenium (brown). Cingulum (light brown), inferior longitudinal fasciculus (dark purple), corticospinal tract (orange), uncinate fasciculus (light blue), inferior fronto-occipital fasciculus (pink). Arcuate fasciculus: direct segment (red), anterior segment (green), posterior segment (yellow).

To compare the behavior of CeFFC algorithm to the FFClust, we applied a bundle segmentation method (Labra et al., 2017; Vázquez et al., 2019) to the fibers obtained by the FFClust and CeFFC clustering algorithms. The segmentation method is based on a multi-subject bundle atlas of deep white matter bundles (see Figure 10). The method labels the fibers that belong to a known bundle, based on a variation of *d*_*ME*_ distance and a defined maximum distance threshold for each bundle. We applied FFClust and CeFFC on 10 subjects for this analysis. First, we applied the segmentation on the fibers obtained by the clustering algorithms. For each bundle in the atlas, we count the number of fibers (*n*_*f*_) and the number of clusters (*n*_*f*_). To evaluate the differences between methods, we calculated the Relative Mean Absolute Difference (RMAD) of the mean *n*_*cl*_ for the 10 subjects ($\bar{n_{cl}}$) and the mean *n*_*f*_ for the 10 subjects ($\bar{n_{f}}$). Table 2 shows that the RMAD of $\bar{n_{cl}}$ if of 5%, with total means almost equals for the number of clusters (343.9 vs 343.7). Also, the RMAD of $\bar{n_{f}}$ is very low, equal to 2.6%, where the total means of the number of fibers are similar, but with a slightly higher value for CeFFC (8315.4 vs. 8521.5). This result is an advantage since CeFFC filters out less meaningful fibers. Finally, we computed the mean of the number of fibers per cluster ($\bar{n_{f}/cl}$) for each bundle. We found that the RMAD of $\bar{n_{f}/cl}$ is only 1.3%, which shows that both methods obtain similar results.

**Table 2:** Bundle segmentation results.

**Fascicle**	**$\bar{n_{cl}}$ FFClust**	**$\bar{n_{cl}}$ CeFFC**	**RMAD $\bar{n_{cl}}$ (%)**	**$\bar{n_{f}}$ FFClust**	**$\bar{n_{f}}$ CeFFC**	**RMAD $\bar{n_{f}}$ (%)**	**RMAD $\bar{n_{f}/cl}$ (%)**
Arcuate fasciculus (LA)	288.7	301.3	4.4	7175.3	7262.7	1.2	0.8
Arcuate fasciculus (RA)	391.5	378.5	3.3	11430.4	11554.6	1.1	1.3
Arcuate fasciculus (LD)	374.3	384.3	2.7	7461.9	7731.8	3.6	0.2
Arcuate fasciculus (LP)	156.3	181.8	16.3	3941.5	3950.9	0.2	3.5
Arcuate fasciculus (RP)	141.4	148.4	5.0	3462.2	3461.5	0.0	1.2
Arcuate fasciculus (RD)	179.4	192.5	7.3	2937.2	3034	3.3	0.6
Corpus callosum (Body)	1051.3	979.5	6.8	43208.2	43831.2	1.4	3.6
Corpus callosum (Genu)	470.0	459.5	2.2	12507.9	12720.5	1.7	1.1
Corpus callosum (Rostrum)	220.7	224.1	1.5	4736.6	4841.4	2.2	0.1
Corpus callosum (Splenium)	279.5	273.3	2.2	7672.4	7884.0	2.8	1.3
Inferior fronto occipital (L)	501.4	520.1	3.7	7581.2	8075.3	6.5	0.4
Inferior fronto occipital (R)	516.7	509.4	1.4	8627.4	9068.9	5.1	1.1
Inferior longitudinal (L)	452.2	468.3	3.6	6304.2	6591.2	4.6	0.2
Inferior longitudinal (R)	579.5	554.6	4.3	8453	8917.6	5.5	1.5
Uncinate fasciculus (L)	215.4	237.6	10.3	4331.3	4431.4	2.3	1.4
Uncinate fasciculus (R)	222.9	231.9	4.0	4653.6	4779.6	2.7	0.3
Corticospinal tract (L)	98.0	90.6	7.6	4447.0	4472.4	0.6	4.0
Corticospinal tract (R)	29.2	30.7	5.1	440.6	444.8	1.0	0.6
Mean	343.9	343.7	5.0	8315.4	8521.5	2.6	1.3

*Comparison of the mean number of clusters $\bar{n_{cl}}$ and the mean number of fibers $\bar{n_{f}}$ between FFClust and CeFFC algorithms. Also, the Relative Mean Absolute Difference (RMAD) of $\bar{n_{cl}}$, $\bar{n_{f}}$ and the mean number of fibers per cluster $\bar{n_{f}/cl}$ is provided. L, Left; R, Right; A, Anterior segment; P, Posterior segment; D, Direct segment*.

Figure 10 shows the segmented bundles for a subject. In Figure 10A it can be seen that the results are visually similar for both methods. Figure 10B shows the segmented bundles for CeFFC on separated displays, where we observe a correct appearance of these deep white matter bundles.

## 4. Conclusion

This work proposes GPGPU parallel algorithms for the state-of-the-art fiber clustering algorithm FFClust (Vázquez et al., 2020). Having a fast fiber clustering algorithm is desired in brain imaging studies, including visualization tools, and applications that need the processing of many subjects such as inter-subject clustering, bundle atlas constructions, and connectivity-based parcellations. The parallel algorithms follow the same steps defined in FFClust, and they can improve the execution time for each step. Step 1 exploits fine-grained parallelism using a GPU. The proposed parallel alternatives for Step 1 of the algorithm uses Kmeans++ method. In addition, this work proposes a variation of the kmeans++ algorithm that aims to avoid selecting outliers as initial centroids. The parallel algorithms for this step use the MAP and Reduce parallel patterns and memory optimizations based on coalescing memory, constant and shared memory on the device. The parallelism of this step provides the highest speedup, which is about 80. The parallelization of the other three steps of FFClust uses the multicore architecture using OpenMP, the parallel thrust library, and a fast algorithm for listing maximal cliques. The overall speedup of the complete parallel method is about 11.5, being able to process a tractography dataset of one million fibers in 3.5 s. The quality of the resulting clusters is about the same as FFClust measured in terms of the DB index metric.

## Data Availability Statement

The datasets generated for this study and source code are available at: http://www.inf.udec.cl/~chernand/sources/CeFFC/.

## Ethics Statement

The studies involving human participants were reviewed and approved by Comité de Protection des Personnes Ile-de-France VII CPP100002/CPP100022, France. The patients/participants provided their written informed consent to participate in this study.

## Author Contributions

IG and PO designed and implemented the main code and experiments. AV implemented the FFClust algorithm and provided analysis codes. CR implemented validations of the methods. CP and J-FM provided the pre-processed ARCHI database. PG supervised the work, provided funding, and wrote the manuscript. CH designed the main research idea, provided guidance on the implementation and evaluation of the algorithms, performed validation analysis, and wrote the manuscript. All authors read and approved the final manuscript.

## Conflict of Interest

The authors declare that the research was conducted in the absence of any commercial or financial relationships that could be construed as a potential conflict of interest.

## Publisher's Note

All claims expressed in this article are solely those of the authors and do not necessarily represent those of their affiliated organizations, or those of the publisher, the editors and the reviewers. Any product that may be evaluated in this article, or claim that may be made by its manufacturer, is not guaranteed or endorsed by the publisher.

## Footnotes

^1^https://networkx.org/documentation/stable/reference/algorithms/generated/networkx.algorithms.clique.find_cliques.html.
